# Effects of Lysozyme-Supplemented Diets on Muscle Texture and Metabolite Profiles in Yellowfin Seabream (*Acanthopagrus latus*)

**DOI:** 10.1155/anu/9977656

**Published:** 2025-11-19

**Authors:** Wenmeng He, Genmei Lin, Lu-jing Gan, Min Chen, Yinjun Ye, Huixin Zhao, Ying Wang, Jianbo Yao, Xuan Cao, Kaidiriye Kaisaier, Kaizhuo Cai, Yitao Zhou

**Affiliations:** ^1^Department of Life Science, Faculty of Science and Technology, Beijing Normal-Hong Kong Baptist University, Zhuhai, Guangdong, China; ^2^School of Marine Sciences, Sun Yat-sen University, Zhuhai, Guangdong, China; ^3^Southern Marine Science and Engineering Guangdong Laboratory (Zhuhai), Zhuhai, Guangdong, China; ^4^Department of Food Science and Engineering, School of Materials and Environment, Beijing Institute of Technology University, Zhuhai, Guangdong, China; ^5^Key Laboratory of Geriatric Nutrition and Health, School of Food and Health, Beijing Technology and Business University, Beijing 100048, China

**Keywords:** flesh muscle, lysozyme-supplemented diets, polyunsaturated fatty acids, untargeted metabolism, yellowfin seabream (*Acanthopagrus latus*)

## Abstract

Yellowfin seabream (*Acanthopagrus latus*) was basal fed supplemented with low (Mus1: 100 mg/kg) and high (Mus2: 200 mg/kg) doses of lysozyme (200 U/mg) diets, compared to a control group (Mus3: basal fed with no lysozyme) to evaluate lysozyme as an antibiotic alternative. Comprehensive analyses (composition, texture, histological, and untargeted metabolomics) revealed lysozyme promoted growth, muscle development, and flesh quality. Lysozyme supplementation enhanced ash and carbohydrate accumulation significantly (*p*  < 0.05). Fish in the Mus1 group showed larger muscle fibers and increased myotome density. Metabolomics identified significant shifts in organic acids, lipids, and aromatic compounds. Mus2 enhanced mucosal immunity and retinal accumulation, while reducing fat deposition. Mus1, with a lower lysozyme dose, showed enrichment of the tricarboxylic acid cycle (TCA) cycle activity, accumulating beneficial organic acids (citric and malic acid) and nutritionally critical fatty acids (EPA and DHA), improving muscle quality. This study provides valuable insights into the effects of dietary lysozyme supplementation on yellowfin seabream, with potential applications for optimizing aquaculture practices and identifying key biomarkers for fish health and growth to improve muscle quality and flavor.

## 1. Introduction

Fish is a food source with high nutritional value. The production and consumption of fish have been increasing during the last few years [1]. Yellowfin seabream (*Acanthopagrus latus*), a euryhaline and omnivorous bottom fish, has yellow pelvic, anal, and caudal fins, long been considered a single valid Indo-West Pacific Ocean species [2]. It is widely distributed and cultured in tropical and subtropical areas such as the Red Sea, Japan, Korea, and the South China Coast [3]. Owing to its strong adaptability to the tough environment, as well as the high quality and good taste of its meat made it both commercially and ecologically important [2, 4]. Yellowfin seabream was considered a good alternative to shrimp culture and a promising candidate for extending marine cage culture [5]. Artificial breeding and cultivation techniques have been well-established for yellowfin seabream in China [6]. In the aquaculture industry, the main issue is disease outbreak [7]. Considering food safety and environmental security, antibiotics are commonly used in aquaculture to prevent disease problems of fish [8]. However, the effects of lysozyme-supplemented diets on meat quality and flavor formation are not clear.

Lysozyme is a natural endogenous antibiotic that induces specific and nonspecific immune responses [9, 10]. As an important antimicrobial protein, it exists in most organisms and plays key roles in protection. Lysozyme may become a potential alternative to antibiotics for the feed industry and be used in different animal studies as a substitute for antibiotics in feed [11]. Information on lysozyme used as a feed additive in aquaculture is limited. It was reported in aquatic animals such as crucian carp, and Pacific white shrimp that lysozyme can improve their growth and immunity [11]. It has also been proven that the lysozyme diet of fish could enhance its growth, cellular immune responses, disease resistance, intestinal microbiome, and morphology, as well as cytokines gene expression in fish [9]. Despite these benefits of dietary lysozyme, the effects of lysozyme-supplemented diets on the flesh quality of yellowfin seabream are rarely reported. The growing demand for high-quality flesh products causes aquaculture farms to pay more attention to improving the flesh quality of farmed fish which will be the major goal of the aquaculture industry in the foreseeable future [12]. Fish muscle texture is affected by physical factors including species, age, and size of the fish within the species, and nutritional status, including water content and distribution, fat content and distribution, and collagen content [8, 13]. Various dietary formulations in aquaculture may significantly impact the nutritional profile and sensory attributes of fish flesh [14]. Thus, alternative feed formulations using lysozyme may also influence the flesh quality of yellowfin seabream.

The flesh quality of fish becomes increasingly important to the industry and directly determines the fish's economic value [15]. The quality of flesh is a complex trait influenced by external and internal factors such as diet, temperature, culture density, and exercise [16, 17]. The safety aspects, nutritional compositions, and sensory properties determine the quality of fish flesh [18]. The skeletal muscle of fish represents a major edible portion exhibiting a diversity of shapes, sizes, anatomical locations, and physiological functions [19]. The fish muscle components include muscle fibers, surrounding intramuscular connective tissue (IMCT), and intramuscular fat (IMF) determining fish flesh quality [12, 20]. Muscle fibers account for 90% of the skeletal muscle, whereas connective and fat tissues account for 10% [19]. The texture is an important standard for evaluating fish flesh quality, as it is a sensory property that determines consumers' perception and satisfaction [12]. The mechanical, structural, and surface properties influence the mouthfeel of meat mouthfeel (oral texture) during chewing [12]. The oral texture is reflected by primary texture properties including hardness (firmness), adhesiveness, cohesiveness, and springiness, and secondary texture properties including chewiness, gumminess, and fracturability [21]. Among the texture attributes, hardness is a vital parameter of fish freshness related to the human-visible acceptability of fish products [18]. In addition, the texture is a sensory interpretation and expression of the interior construction or structure of flesh. Fish structure and microstructure measurements are also vital for further understanding fish flesh quality [18]. The measurement of texture methods uses sensory and instrumental measurements such as texture profile analysis (TPA), whereas optical and electron microscopy are used to determine the structure and microstructure of fish flesh [18]. Texture determination microscopy is an effective measurement tool [18].

Metabolomics, a high-throughput analysis method, is increasingly being applied in aquaculture research to characterize and quantify biomolecules. This approach reflects organismal responses to both intrinsic and extrinsic factors, providing crucial insights into the biological states of the studied organisms [22]. Additionally, metabolomics offers a new strategy for food quality assessments, as metabolites play a crucial role in determining fish quality, including flavor and aroma characteristics, nutritional value, and degradation processes [23]. In particular, metabolites such as carbohydrates, proteins, organic acids, nucleotides, and their derives are the main taste-producing components in fish flesh [24]. This process contributes to the development of flavor in fish muscle, with the continuous accumulation of these precursors enhancing both flavor intensity and muscle quality [17, 24]. In addition, the analyses of the metabolic pathways of targeted metabolites identified through metabolomics studies of flavor and nutritional components can help regulate fish feeding, thereby improving the quality of fish flesh [17].

To consistently produce high-quality flesh products from yellowfin seabream, understanding the muscle components and their correlations with texture properties under lysozyme-supplemented diets is crucial. This investigation aims to evaluate and compare the proximate composition of three experimental feed groups with varying lysozyme concentrations and a control group; assess and compare texture profiles and microstructural characteristics of fish muscle; and apply metabolomics to comprehensively analyze molecular mechanisms underlying lysozyme's impact on muscle quality.

## 2. Materials and Methods

### 2.1. Ethical Procedures

All experimental procedures in this study with yellowfin seabream (*Acanthopagrus latus*) were approved by the Ethics Committee of Sun Yat-sen University (Protocol No. SYSU-IACUC-2025-B1028; Period of Protocol: 2023.01.01-2027.12.31; Approval Date: 2025.04.01), and the methods were carried out following the approved guidelines.

### 2.2. Fish Samples

One-year-old male yellowfin seabream was collected from a local fish farm (Zhuhai, China) in November 2023, with a body weight of 70.2 ± 4.6 g and a body length of 12.1 ± 0.4 cm. The fish were first acclimated to laboratory conditions for 10 days using a basal feed under the following conditions: salinity 4.91 ± 0.24 ppt, temperature 23.2 ± 0.5°C, and a 14 h/10 h light–dark photoperiod, with automated water circulation and continuous gentle aeration. The basal feed formula included crude protein (≥ 44%), crude fat (≥4%), crude fiber (≤5%), crude ash (≤ 15%), and lysine (≥2.5%), with moisture content not exceeding 11%. After acclimation, fish were randomly divided into three groups with triplicate tanks per group, namely Mus1: basal feed supplemented with 100 mg/kg lysozyme; Mus2: basal feed supplemented with 200 mg/kg lysozyme; Mus3: control group (basal feed with no lysozyme). The lysozyme product containing 200 U lysozyme per mg powder was purchased from Zhejiang Aegis Biotech Co., Ltd., and coated the feed before usage. During the feeding trail, fish were fed their respective diets twice daily (3% of total body weight per day) until 1 day before sampling. After 4 weeks, nine fish per group were euthanized with tricaine methane sulfonate (MS-222, 200 mg/L) and sampled. Muscle tissues were collected and stored at 4°C.

### 2.3. Composition Determination

According to the method reported by Wang et al. [25], moisture content was determined using the direct drying method specified in GB5009.3-2016 (GB: National Standard of China). Fish samples were dried at 105°C until reaching constant weight (typically 4–6 h, with ≤2 mg weight change between successive weighing). Kjeldahl nitrogen, including digestion, distillation, and titration specified in GB5009.5-2016 was used to evaluate the crude protein content in fish flesh. Crude fat content was determined via Soxhlet extraction (GB5009.6-2016). The Section 2.2.8 of GB/Z21922-2008 was used to analyze the carbohydrate content. Finally, ash content was quantified using the muffle furnace burning method (GB5009.4–2016). The detailed procedure description was followed, as reported by Zhang et al. [26]

### 2.4. TPA

TPA is a critical method for assessing the sensory quality of fish. Fish back muscle was cut into 2 cm × 2 cm × 2 cm cubes to uniform-sized muscle cubes. The samples were analyzed using a texture analyzer (EZ-SX 500 N, Shimadzu, Japan) equipped with a 500 N load cell, TRAPEZIUM X software, an upper cylindrical press jig (ø36 mm, plastic), and a lower compression plate (ø118 mm, stainless steel). The testing mode of TPA was set with a 30% deformation level of the original sample height. Also, a 10 g trigger force was used with a 2 s wait time between compressions. The pretest and posttest speeds were both set at 2 mm/s, the test speed was set to 5 mm/s. In the TPA textural measurements, five parameters of the samples were recorded: hardness (peak force of the first compression cycle), cohesiveness (ratio of positive force area during the second compression compared to that during the first compression cycle), adhesiveness (negative force area under the baseline between the compression cycle), springiness (height that the food recovers during the time elapsing between the two compression cycles), and chewiness (hardness multiplied by cohesiveness multiplied by springiness). For each group of Mus1, Mus2, and Mus 3 (Control), six replicate samples were collected and analyzed [27, 28].

### 2.5. Transmission Electron Microscopy (TEM)

All procedures involving chemical fixatives (including TEM fixative and osmium tetroxide) were performed in a fume hood with appropriate personal protective equipment (PPE; gloves, lab coat, and eye protection) in accordance with standard laboratory safety practices.

The fresh fish tissue was harvested and immediately placed in Petri dishes containing TEM fixative. The tissue was then cut into 1 mm^3^ pieces within the fixative. The tissue blocks were transferred into a microcentrifuge tube with fresh TEM fixative for further fixation at 4°C, followed by preservation and transportation. Subsequently, the tissues were washed three times (15 min each) with 0.1 M phosphate buffer (PB; pH 7.4). All subsequent steps were performed in the dark. The tissues were postfixed with 1% osmium tetroxide (OsO4) in 0.1 M PB (pH 7.4) for 2 h at room temperature. After OsO4 removal, the tissues were rinsed three times (15 min each) with 0.1 M PB (pH 7.4). Dehydration was carried out at room temperature using a graded ethanol series (30%, 50%, 70%, 80%, and 95%) for 20 min each, followed by two changes of 100% ethanol (20 min each). Finally, the tissues were treated with two changes of acetone (15 min each).

For rinse preparation, tissue samples were incubated in a 1:1 mixture of acetone and Epoxy Embedding Medium 812 (EMBed 812) at 37°C for 2–4 h, and then a 1:2 acetone/EMBed 812 mixtures overnight at 37°C, as well as pure EMBed 812 for 5–8 h at 37°C.

The tissues were embedded in pure EMBed 812 within embedding molds and cured overnight at 37°C. The molds were then transferred to a 60°C oven for polymerization (> 48 h). Resin blocks were sectioned into 1.5 μm slices using a semi-thin microtome, stained with toluidine blue, and examined under a light microscope for orientation. Selected areas were trimmed and sectioned to 60–80 nm thickness using an ultramicrotome. Sections were mounted onto 150-mesh copper grids coated with formvar film. Grids were stained with 2% uranyl acetate in saturated ethanol (8 min, dark), rinsed with 70% ethanol (three times) and ultrapure water (three times). Lead citrate staining (2.6%, 8 min) was conducted in a CO_2_-free environment, followed by ultrapure water rinses (three times). After drying with filter paper, grids were stored in a grid storage board overnight at room temperature. Imaging was performed using a Hitachi HT7800 transmission electron microscope equipped with a Hitachi TEM imaging system.

### 2.6. Histological Observation

Fish muscle tissue was processed according to the standard operating procedure (SOP) of Servicebio (Wuhan Servicebio Technology Co., Ltd.) including pathological tissue sampling, fixation, and paraffin embedding. Paraffin sections (about 3 μm) were sequentially immersed in environmentally friendly dewaxing (G1128, Servicebio)/transparent liquid I (20 min), dewaxing/transparent liquid II (20 min), anhydrous ethanol I (5 min), anhydrous ethanol II (5 min), 75% ethanol (5 min), and running water for rinsing. Frozen sections (about 3 μm) were thawed to room temperature from −20°C, fixed with tissue fixative (G1101, Servicebio, 15 min), and rinsed with running water. Sections were pretreated with Hematoxylin–Eosin (H&E) HD constant dye kit (G1076, Servicebio) staining solution (1 min), stained with Hematoxylin solution (5%, 3–5 min), rinse with pure water, and treated with Hematoxylin differentiation solution and Hematoxylin bluing solution, followed by rinsing. Sections were dehydrated in 95% ethanol (1 min), counterstained with Eosin for 15 s, and sequentially immersed in absolute ethanol I, II, III (2 min each), n-butanol I, II (2 min each), xylene I, II (2 min each). Sections were mounted with neutral gum and imaged using a pannoramic scanner and analyzed with CaseViewer 2.4 software.

### 2.7. Untargeted Metabolomics

Fresh fish muscle tissue samples were snap-frozen in liquid nitrogen, freeze-dried, and stored at −80°C for further analysis. A 0.5 g aliquot of each freeze-dried tissue placed into a 2 mL centrifuge tube with 400 µL of extraction solvent (methanol:water = 4:1, v:v) and 0.02 mg/mL internal standard (L-2-chlorophenylalanine) for metabolites extraction. The mixture was ground using a tissue grinder (6 mm diameter) at −10°C and 50 Hz for 6 min. After ultrasonic extraction at 5°C and 50 kHz for 30 min, the sample was incubated at −20°C for 30 min to facilitate metabolite extraction, followed by centrifugation for 15 min at 13,000 *g* and 4°C. The supernatant was then transferred to sample vials with inserts for further LC–MS analysis using an AB SCIEX UHPLC-Triple TOF system with an ACQUITY UPLC HSS T3 column (100 mm × 2.1 mm i.d., 1.8 µm; Waters, Milford, USA). Chromatographic conditions were as follows: mobile phase A consisted of 95% water and 5% acetonitrile (0.1% formic acid), mobile phase B consisted of 47.5% acetonitrile, 47.5% isopropanol, and 5% water (0.1% formic acid). The flow rate was 0.40 mL/min with an injection volume of 10 µL. The column temperature was maintained at 45°C. Quality control (QC) samples were prepared by pooling equal-volume aliquots from all experimental sample extracts. Each QC sample matched the volume of individual experimental samples and underwent identical processing and analysis protocols. During instrumental analysis, QC samples were analyzed after every 5–15 experimental samples to monitor analytical stability.

Mass spectrometry analysis was conducted using electrospray ionization (ESI) in both positive and negative ion modes, with a scan range of m/z 50–1200. Key instrumental parameters included nebulizer gas (50 psi), auxiliary gas (50 psi), curtain gas (35 psi), ion source temperature (500°C), ion spray voltages (± 5500 V), delustering potential (80 V), and collision energy (20–60 eV). Raw data were processed via Progenesis QI v3.0 software for baseline filtering, peak detection, integration, retention time correction, and alignment, generating a data matrix of retention times, m/z values, and peak intensities. Metabolites were identified by matching MS/MS spectra against HMDB, METLIN, and in-house databases, with a mass error threshold of <10 ppm and validation through fragment ion consistency. Collected data were analyzed by performing principal component analysis (PCA), partial least squares discriminant analysis (PLS-DA) to distinguish their metabolites differences among fish groups. By comparison, metabolites with variable importance in projection (VIP) > 1 and adjusted *p* < 0.05 were considered significantly differentially metabolites. Subsequently, differential metabolites were mapped into their biochemical pathways through metabolic enrichment and pathway analysis based on Kyoto Encyclopedia of Genes and Genomes (KEGG) database. For each group of Mus1, Mus2, and Control, six replicate samples were collected and analyzed.

### 2.8. Statistical Analysis

Data are presented as mean ± standard deviation. One-way ANOVA with homogeneous variance assumptions was performed using SPSS 22.0 (SPSS Inc., Chicago, USA) at a significance level of *p*  < 0.05. Homogeneity of variances was confirmed via Levene's test.

Additional analyses including PCA, PLS-DA, and data visualization (Vocalnol, Venn, Heatmap, and KEGG diagrams), were conducted using the Majorbio Cloud Platform (https://www.majorbio.com/web/www/index).

## 3. Results and Discussion

### 3.1. Proximate Composition of Fish Flesh

As shown in Table 1, ash, crude protein, crude fat, and total carbohydrate levels in muscle tissue increased significantly in fish fed lysozyme-supplemented diets (Mus1: 100 mg/kg; Mus2: 200 mg/kg) compared to the unsupplemented Mus3 (control group:basal diet only). However, only ash content in Mus1 (2.97 ± 0.06 g/100 g) and Mus2 (3.27 ± 0.06 g/100 g) showed significant elevation (*p*  < 0.05) compared to Mus3 (2.83 ± 0.06 g/100 g), suggesting that the dietary lysozyme may enhance mineral retention in fish muscle. Additionally, moisture content was marked lower in Mus1 and Mus2 than in the control group.

Proximate composition indices are widely recognized as reliable markers for assessing the nutritional quality and value of fish muscle [29]. The reduced moisture content in Mus1 and Mus2 correlated with higher fat, protein and energy density compared to Mus3, aligning with findings reported by Ahmed et al. [29] and Yıldırım et al. [30]. Furthermore, the observed compositional profile supports the classification of yellowfin seabream (*Acanthopagrus latus*) as low-lipid (less than 5%) and high-protein (about 20%) species [31]. These results indicate that the lysozyme supplementation likely promotes lipid and protein deposition in yellowfin seabream.

### 3.2. TPA of Fish Flesh

As shown in Table 2, seven key texture attributes of fish muscles were determined. Those attributes are critical determinants of commercial value and processing suitability of fish muscle [32]. The hardness of lysozyme-supplemented diets groups (Mus1: 23.82 ± 14 N and Mus2: 28.36 ± 11 N) was significantly higher (*p* < 0.05) than the control group (Mus3: 13.18 ± 10 N). Similarly, adhesiveness (force required to compress food between the molars), and gumminess (energy needed to disintegrate semisolid food for swallowing) in Mus1 and Mus2 showed significant increases compared to Mus3, likely due to lysozyme supplementation [12]. Dietary lysozyme may enhance flesh quality through modulation of gut health and nutrient metabolism, and immune parameters [9]. In contrast, chewiness (energy required to chew solid food for swallowing) did not differ significantly among groups. Consistent with proximate composition data (Section 3.1), Mus1 and Mus2 exhibited elevated ash content, which correlates with muscle texture and flavor [33]. As reported by Wang et al. [12], higher firmness indicates superior fish muscle quality, suggesting that lysozyme-enhanced diets improved muscle quality in Mus1 and Mus2 relative to the control group (Mus3).

### 3.3. Histological Observation of Fish Muscle Fiber

**Figure 1:**
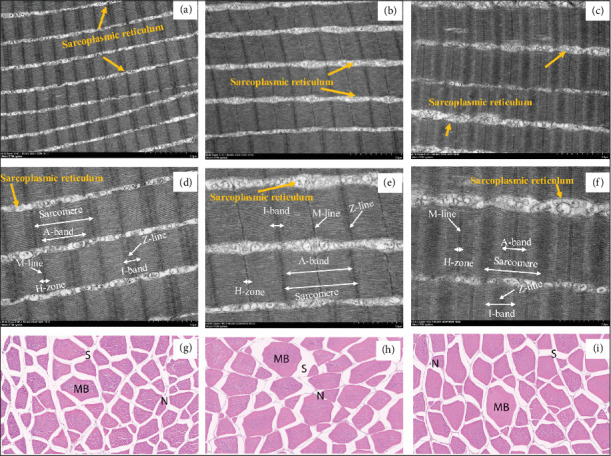
Transmission electron micrographs and photomicrographs of muscle fibers in yellowfin seabream (*Acanthopagrus latus*) fed experimental diets. *Note:* 1. (a–c) Transmission electron micrographs (TEM) of longitudinal sections of white muscle. Scale bar: 2 μm. 2. (d–f) TEM of longitudinal sections of white muscle. Scale bars: 1 μm. 3. (g–i) Photomicrographs of transverse sections of white muscle. Scale bars: 20 μm. 4. 1M group (a, d, g): Fed basal diet supplemented with 100 mg/kg lysozyme. M2 group (b, e, h): Fed basal diet supplemented with 200 mg/kg lysozyme. 3M group (c, f, i): Control group fed basal diet without lysozyme.

Structural changes in muscle proteins influence meat quality [34]. These proteins are organized into striated muscles fibers [35]. The sarcomere is the smallest contractile unit serving as the foundational structure [36]. Transmission electron micrographs of longitudinal sections from lysozyme-supplemented diets yellowfin seabream (*Acanthopagrus latus*) are shown in Figure 1a–f (scale bars were set as 2 μm and 1 μm). As shown in Figure 1, the sarcomere is a highly organized structure bounded by Z-lines (containing α-actinin and associated proteins) [34] and features A-bands (thick myosin-actin filaments), I-bands (thin actin filaments) [37], and H-zones (lighter regions centered by M-lines rich in myomesin) [37]. Disruption in I-band integrity reduces muscle structural stability [38]. Sarcoplasmic reticulum (SR), visible as circular particles, surrounds myofibrils interconnected by endomysial connective tissue.

The transmission electron micrographs shown in Figure 1 indicate that the total transverse fiber area of Mus1, Mus2, and Mus3 groups. Compared to Mus2 and Mus3, larger transverse fiber area and higher density of small-diameter fibers were found in Mus1 (100 mg/kg lysozyme fed). Longer sarcomere and A-band but thinner M-lines and I-bands were found in Mus2 than those in Mus1 and Mus3. Additionally, larger Z-disks and SR volumes was found in Mus3 than in Mus1 and Mus2. These results demonstrated that a low-dose lysozyme (Mus1) enhance muscle fibers diameter and myotomes growth, correlating with improved water retention and hardness [39]. Smaller myofiber diameter and higher density in Mus1 align with superior flesh quality and firmness, consistent with texture analysis results.

Furthermore, transverse section analysis of yellowfin seabream white muscle results are shown in Figure 1. Muscle bundles in all groups exhibited polygonal morphology and closely packed in the cross-section and well organized for Mus3 (i), Mus1 (g), and Mus2 (h) [40]. Each irregular muscle bundle contains a variable number of peripheral nuclei or marginal encysted structures, surrounded by loosely organized fibrous tissue [41]. The unstained white space (denoted “S”) represent intermuscular connective tissue [41]. Mus2 muscle fibers exhibited wider unstained space (S) and fewer myofibers compared to in Mus1 and Mus3. No significant differences in these morphological indices were observed, suggesting that myofiber morphology may not be primarily drive texture changes in fish muscle, consistent with findings reported by Wu et al. [42].

### 3.4. Metabolomic Profiles of Fish Muscle Samples

Metabolites from the three experimental fish groups (Mus1, Mus2, and Mus3) were analyzed using LC–MS in positive and negative ion modes using a nontargeted approach. A total of 13,708 metabolites were detected (6643 in negative mode; 7065 in positive mode). Subsequent analysis identified 1902 compounds (938 in negative mode and 964 in positive mode), of which 1814 compounds were confirmed against a reference library, and 993 compounds annotated and mapped to pathways using the KEGG database.

**Figure 2:**
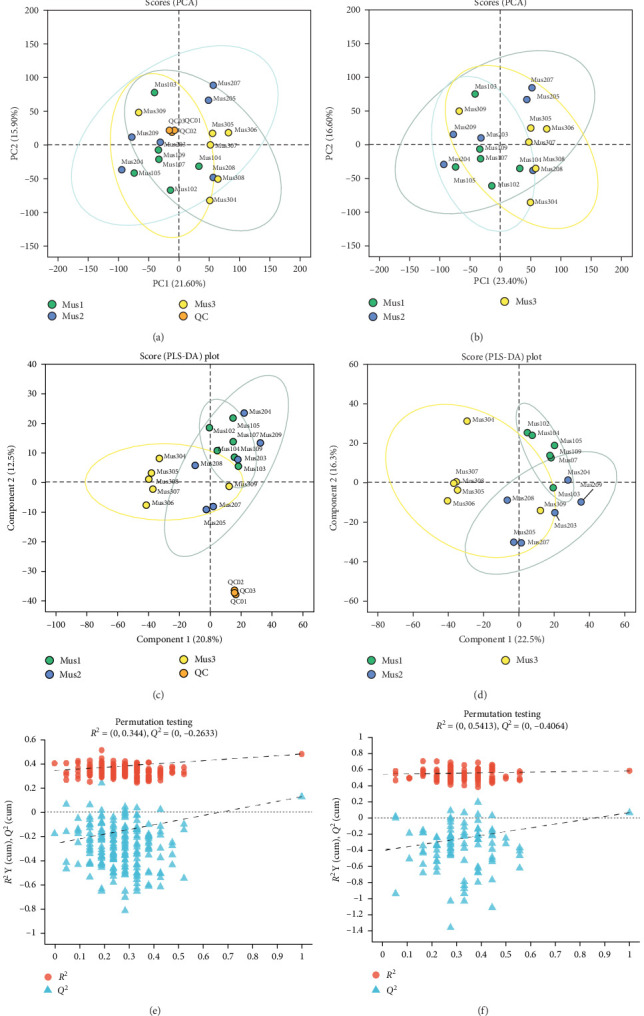
Nonvolatile metabolite profiles in yellowfin seabream (*Acanthopagrus latus*) muscle tissue under experimental diets. (a) Principal component analysis (PCA) of nonvolatile metabolites. including quality control (QC) samples, (b) PCA of nonvolatile metabolites excluding QC samples, (c) partial least squares discriminant analysis (PLS-DA) including QC samples, (d) PLS-DA excluding QC samples, (e) permutation testing of PLS-DA with QC samples, (f) permutation testing of PLS-DA without QC samples. *Note:* The three groups of fish include Mus1 (basal feeds supplemented with 100 mg/kg lysozyme), Mus2 (basal feeds supplemented with 200 mg/kg lysozyme), and Mus3 (basal feeds without lysozyme) designed as a control group.

An unsupervised PCA model was applied to assess the metabolic profile differences among groups. PCA score plots (Figure 2a with QC samples; Figure 2b without QC samples) revealed that QC samples clustered tightly in Figure 2a, indicating method stability and high data quality [24]. After QC removal (Figure 2b), PCA explained 40% total variance (PC1 accounting for 23.4% and PC2 for 16.6%). However, overlapping confidence ellipses suggested poor intergroup discrimination [43].

PLS-DA, a supervised discriminant analysis method, was used to reduce multicollinearity among variables and enhance group differentiation [43]. This analysis further elucidated the metabolomic profiles of fish muscle samples, with QC illustrated in Figure 2c and non-QC samples in Figure 2d. Permutation test results (Figure 2e,f) assessed the model's fit, robustness, and validity [24]. In Figure 2c, the tight clustering of QC samples in the PLS-DA underscores high data quality and reproducibility. Figure 2d demonstrates clear separation among the three fish groups, accounting for 38.8% of the total variance (23.4% PC1, 16.6% PC2). The smallest confidence ellipse for Mus1 indicates superior data reliability and model validity [17]. Permutation tests (Figure 2e,f) depict *R*^2^Y (red dots) and *Q*^2^ (blue dots) values, reflecting model fit (perfect fit = 1) and predictability (ideal *Q*^2^ = 1), respectively [17]. In this investigation, the PLS-DA model achieved an *R*^2^ of 0.5413, indicating moderate fit, while the positive slope of *Q*^2^ confirmed no overfitting [24]. These findings suggest that lysozyme concentrations in the diet influence fish metabolism. Also, [44] reported that artificial diets alter lipid composition and muscle quality in grass carp (*Ctenopharyngodon idella*).

## 4. Differential Metabolites Screening of Fish Muscle Samples

Differential metabolites, including unidentified compounds detected using mixed-mode analytical techniques, were screened using univariate statistical analysis [45]. Significant metabolites were identified based on VIP > 1.5 from orthogonal projections to latent structures discriminant analysis (OPLS-DA), *p*  < 0.05 from Student's *t*-test ([46, 47]). Fold change (FC)-representing the ratio of metabolites abundance between groups—was calculated [48]. Metabolites with FC < 1 (downregulated) or FC > 1(upregulated) were selected. A volcano plot was generated using log_2_(FC) as the *x*-axis and −log_10_ (*p*-value) as the *y*-axis to visualize metabolomic differences.

**Figure 3:**
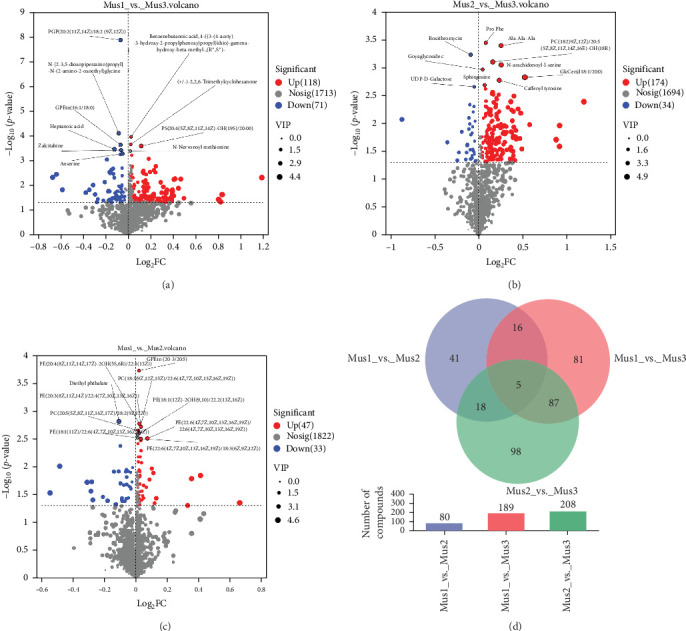
Differential nonvolatile metabolites in yellowfin seabream (*Acanthopagrus latus*) muscle tissue under experimental diets. (a) Volcano plot of differential metabolites between Mus1 and Mus3, (b) volcano plot of differential metabolites between Mus2 and Mus3, (c) volcano plot of differential metabolites between Mus1 and Mus2, (d) venn diagram illustrating differential shared and unique differential metabolites among groups. *Note:* The three groups of fish include Mus1 (basal feeds supplemented with 100 mg/kg lysozyme), Mus2 (basal feeds supplemented with 200 mg/kg lysozyme), and Mus3 (basal feeds without lysozyme) designed as a control group.

Volcano plots illustrating differential metabolites between sample groups are shown in Figure 3a (Mus1 vs. Mus3), Figure 3b (Mus2 vs. Mus3), and Figure 3c (Mus1 vs. Mus2). Red dots represent upregulated metabolites, blue dots denote downregulated metabolites, and gray dots indicate nonsignificant metabolites. Dot size corresponds to VIP values. The top 10 most significant metabolites (ranked by ascending *p*-values) are labeled.

Comparing Mus1 with Mus3 (Figure 3a), Mus1 exhibited 118 upregulated and 71 downregulated metabolites. Key upregulated compounds include benzenebutanoic acid derivatives, cyclic ketone, and hydroxylated phosphatidylserine. Significantly downregulated metabolites include phospholipids (e.g., PGP [20:2/18:2], GPEtn [16:1/18:0]), heptanoic acid, zalcitabine, anserine, and asparaginyl-proline.

When comparing Mus2 with the Mus3 group, Figure 3b reveals that Mus2 exhibited 174 upregulated and 34 downregulated metabolites relative to the Mus3. Key upregulated metabolites in Mus2 include pro-phe, ala-ala-ala, PC (18:2 [9Z, 12Z] /20:5[5Z, 8Z,11Z, 14Z,16E]-OH[18R]), N-arachidonoyl-l-serine, goyaglycoside c, glccer (d18:1/20:0), sphingosine, and caffeoyl tyrosine. In contrast, Roxithromycin and UDP-D-Galactose were significantly downregulated in Mus2.

In both Mus1 and Mus2, significant differences were observed in metabolites related to lipids and lipid-like molecules, organic acids and derivatives, benzenoids, phenylpropanoids and polyketides, nucleosides, nucleotides, and analogs compared to the control group. Mus1 and Mus2 were fed basal diets supplemented with 100 mg/kg and 200 mg/kg lysozyme, respectively. These results suggest that increasing lysozyme concentration in fish feed alters metabolite profiles, particularly enhancing lipids and lipid-like molecules. Additionally, 47 metabolites were upregulated, and 33 downregulated in Mus1 and Mus2, indicating a narrower differential metabolite profile (Figure 3c). Compared to Mus2, the Mus1 group (lower lysozyme concentration) showed significant upregulation of unsaturated lipids, while diethyl phthalate was downregulated.

A Venn diagram (Figure 3d) was generated to compare metabolite differences among the three groups [49]. The largest differences occurred between Mus2 and Mus3, indicating that the Mus2 diet significantly influenced metabolites compared to the control (Mus3). Conversely, differences between Mus1 and Mus2 were minimal, suggesting lysozyme concentration modulates metabolite variation. Five metabolites were commonly altered across all groups.

Notably, lysozyme-supplemented diets fish (Mus1 and Mus2) exhibited upregulated phospholipid compositions muscle, with Mus1 (lower concentration) showing a more pronounced effect. Similar trends were observed in large yellow croaker fed glycerol monolaurate [23]. Additionally, kaempferol supplementation has been reported to alter lipid metabolism [50].

## 5. Differential Metabolites Analysis

**Figure 4:**
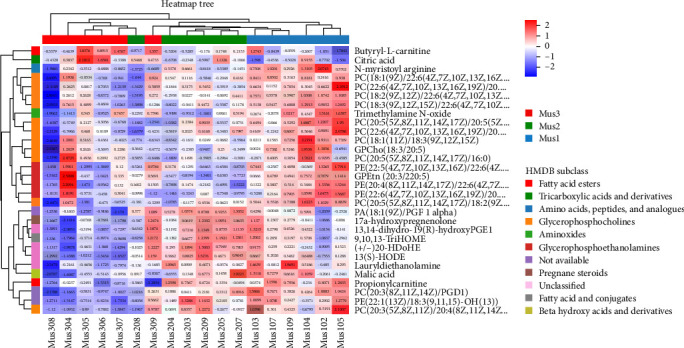
Heatmap of the top 30 selected differential metabolites in yellowfin seabream (*Acanthopagrus latus*) under experimental diets. Hierarchical clustering illustrates metabolite abundance levels across groups (Mus1, Mus2, and Mus3), with red and blue hues indicating high and low abundance, respectively. *Note:* The three groups of fish include Mus1 (basal feeds supplemented with 100 mg/kg lysozyme), Mus2 (basal feeds supplemented with 200 mg/kg lysozyme), and Mus3 (basal feeds without lysozyme) designed as a control group.

The top 30 significantly different metabolites, ranked by VIP values, were analyzed using a heatmap (Figure 4) to illustrated the enrichment of these metabolites in the three groups of fish. The top metabolites included phosphatidylcholine (PC), phosphatidylethanolamine (PE), and phosphatidic acid (PA). Phospholipids comprise sphingomyelin (SM) and glycerophospholipids [51]. Glycerophospholipids consisted of L-glycerol 3-phosphate, fatty acids, phosphate groups, and polar head groups [52], forming PC, PE, PA, phosphatidylserine (PS), phosphatidylglycerol (PG), phosphatidylinositol (PI), and lysophospholipids lysophosphatidic acid (LPA), lysophosphatidylcholine (LPC), and lysophosphatidylethanolamine (LPE) [51]. PC and PE are abundant in aquatic species. Also, they are critical for growth and volatile flavor compound formation [52]. It is known that triglycerides and phospholipids are the major lipids classes in fish and are the substrates for the formation of volatile flavor compounds [53]. The heatmap shown in Figure 4 indicates that Mus1 and Mus2 showed high abundance in PC and PE, while Mus3 was low aligning with higher crude fat content in lysozyme-supplemented diets groups (Mus1, Mus2) shown in Table 1. Muscle fat deposition, especially the unsaturated fatty acids profile is crucial nutritional standard to evaluate high-quality of fish muscle [54]. Lysozyme altered lipid metabolism, particularly glycerophospholipid pathways [55].

Furthermore, citric acid, malic acid, succinic acid, and lactic acid are the principal acid taste-producing agents in seafood [56, 57]. The heatmap shows that citric acid (negatively correlated with Mus1/Mus2) and malic acid (positively correlated) significantly differed among groups, suggesting lysozyme modulates taste by reducing citric acid and accumulating malic acid. Similar mechanisms affect oyster flavor [58].

**Figure 5:**
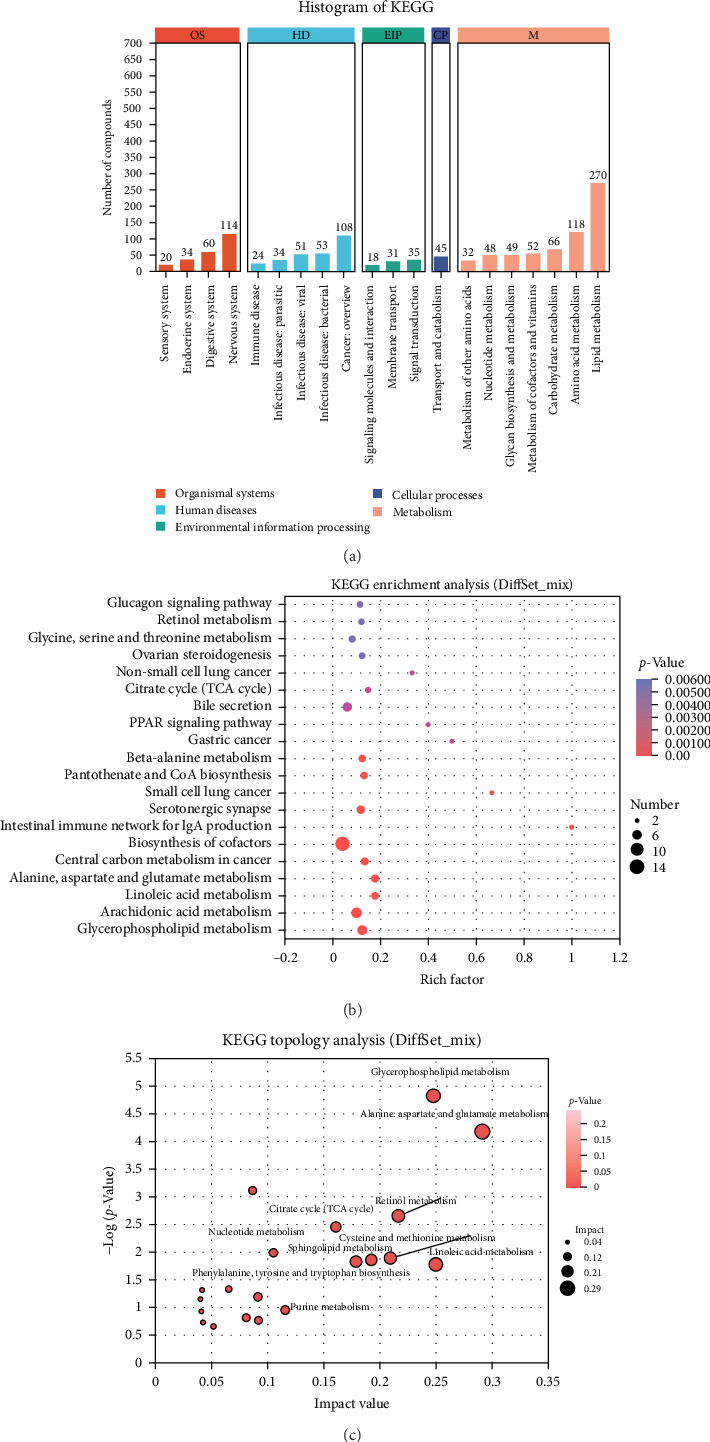
KEGG analysis of the differentially enriched metabolic pathways in yellowfin seabream (*Acanthopagrus latus*) fed experimental diets. (a) KEGG pathways classification in yellowfin seabream muscle tissue, (b) enrichment analysis of the top 20 differentially enriched metabolic pathways, (c) topology analysis of the top 20 differentially metabolic pathways.

Significantly different metabolites were annotated using the KEGG database by matching mass spectrometry data [59]. KEGG pathway analysis (Figure 5a) revealed that metabolism-related pathways—particularly lipid metabolism (270 pathways)—were most enriched. KEGG enrichment analysis is a widely used tool for identifying key metabolic pathways in fish studies, especially for differential metabolites [43]. A bubble plot of the top 20 differential metabolites (Figure 5b) showed that cofactor biosynthesis pathways contained the highest number of differential metabolites, followed by glycerophospholipid metabolism, arachidonic acid (ARA) metabolism, linoleic acid (LA) metabolism, and alanine, aspartate, and glutamate metabolism. Moreover, the KEGG topology was also conducted using point size representing specific pathways' impact value, and the dark red color represents their *p*-values and results shown in Figure 5c. Topology analysis highlights the five most impactful, including “Glycerophospholipid metabolism,” “alanine, aspartate, and glutamate metabolism,” “retinol metabolism,” “cysteine and methionine metabolism,” and “linoleic acid metabolism.” These results demonstrate that lipid and amino acid metabolism pathways were significantly altered across lysozyme-supplemented diets groups. Lipid metabolism involves enzymatic processes such as digestion, absorption, transport, synthesis, and decomposition [60]. Supporting this, LA metabolism, ARA metabolism, and glycerophospholipid metabolism were markedly altered in Mus1 and Mus2 compared to the control (Mus3) (Supporting Information: Figures S1 and S2).

Notably, Mus1 exhibited elevated glycerophospholipids metabolism (Supporting Information: Figures S1a,b; S2a,b; and S3a,b). Mus2 showed enhanced bile secretion (Supporting Information: Figures S1a,b and S2a,b), a biomarker of lipid metabolism [61]. Mus2 displayed upregulation of the intestinal immune network for IgA production (Supporting Information: Figure S2), indicating lysozyme enhances mucosal immunity [62, 63]. This might be the reason that cause increasing anti-inflammatory effects, thus, improved quality and nutrition profile of fish flesh [63, 64]. It is reported that the interleukin-1β (IL-1β) and TNF-α are cytokines paying a pivotal role in the regulation of the systemic inflammatory response and immune function [63]. In addition, retinol metabolism was significantly enriched in Mus2 (Supporting Information: Figure S3b). Mus2 exhibited lower crude fat content compared to Mus1 and Mus3. This finding could relate to the role of retinol (vitamin A), which in certain systems reduces fat deposition by inhibiting lipid mobilization [54], noting that such effects may be species-specific and dose-dependent (Table 1). In conclusion, low-dose lysozyme (Mus1) promotes glycerophospholipid accumulation. High-dose lysozyme (Mus2) enhances mucosal immunity, retinol metabolism, and bile secretion while reducing fat deposition.

### 5.1. Pathways of Key Metabolites

**Figure 6:**
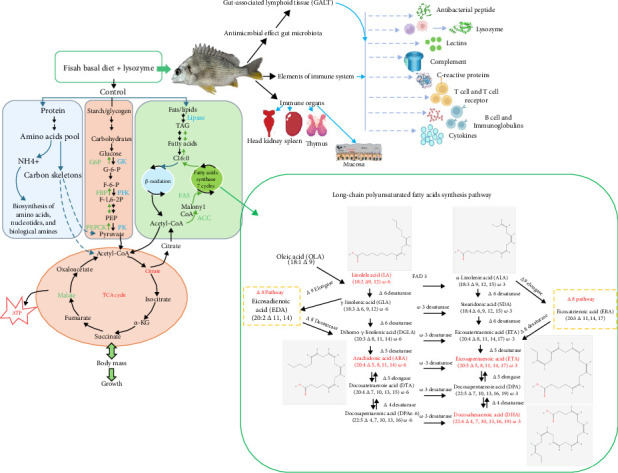
Key metabolic pathway of targeted metabolites and immune system in yellowfin seabream (*Acanthopagrus latus*).

As discussed in previous sections in this investigation, citric acid and malic acid are critical intermediates in the tricarboxylic acid cycle (TCA). They showed significant differences among lysozyme-supplemented diets groups (Mus1, Mus2) and the control group (Mus3) (Figure 6). Citric acid, the initial TCA cycle metabolite, was lower in Mus1 and Mus2 (Figure 4), while malic acid, a later-stage intermediate, exhibited an increased amount in the two groups. These findings suggest lysozyme enhances energy production (via ATP synthesis) and malic acid accumulation in the TCA cycle, particularly in the low-dose lysozyme group (Mus1) (Supporting Information: Figure S1a).

For long-chain polyunsaturated fatty acids (LC-PUFAs) synthesis, Acetyl-CoA from the TCA cycle initiates LC-PUFA biosynthesis, producing eicosapentaenoic acid (EPA) (C20:5 Δ5,8,11,14,17) and docosahexaenoic acid (DHA) (C22:6 Δ4,7,10,13,16,19) (Figure 6). Marine fish, including yellowfin seabream, synthesize these omega-3 fatty acids via aerobic pathways involving desaturases and elongases [65]. Oleic acid (OLA, C18:1 Δ9) is synthesized in plastids via fatty acid synthase (FAS). OLA (C18:1 Δ9) is desaturated to LA (C18:2 Δ9,12, ω-6 pathway) or α-linolenic acid (ALA, C18:3 Δ9,12,15, ω-3 pathway), as shown in Figure 6 [65].

For EPA (C20:5 Δ5,8,11,14,17) synthesizing via the ω-6 pathway, LA is subsequently converted to γ-linolenic acid (GLA, C18:3 Δ6,9,12) catalyzed by Δ6 desaturase, to di-homo-γ-linolenic acid (DGLA) (C20:3 Δ8,11,14) catalyzed by Δ6 elongase, to ARA (C20:4 Δ5,8,11,14) catalyzed by Δ5 desaturase and then converted to EPA by Δ3 desaturase. In the ω-3 pathway, ALA (C18:3 Δ9,12,15) is subsequently converted to stearidonic acid (SDA, C18:4 Δ6,9,12,15), eicosatetraenoic acid (ETA, C20:4 Δ8,11,14,17), and finally synthesize EPA through Δ6 desaturase, Δ6 elongase, and Δ5 desaturase, respectively.

Regarding the formation of DHA (C22:6 Δ4,7,10,13,16,19) via the ω-6 pathway based on the previous formation of ARA, then ARA is catalyzed by Δ5 elongase to form docosatetraenoic acid (DTA, C22:4 Δ7,10,13,16), desaturating by Δ4 desaturase to form docosapentaenoic acid (DPA*n*−6, C22:5 Δ4,7,10,13,16) and then converts to DHA via ω-3 desaturase. In the other ω-3 pathway based on the formation EPA, then the EPA converts to DPA (C22:5 Δ7,10,13,16,19) by Δ5 elongase, and then DPA is desaturated by Δ4 desaturase to finally form DHA.

Lysozyme-supplemented diets groups (Mus1, Mus2) showed elevated LA and ARA levels (Figure 5b, Supporting Information: Figures S1a, S2a), precursors for EPA/DHA biosynthesis. Mus1 (100 mg/kg lysozyme) accumulated more unsaturated fatty acids (Figure 4) and exhibited higher PE (22:6/22:6) levels (Supporting Information: Figure S3c), suggesting greater DHA incorporation compared to Mus2 (200 mg/kg lysozyme). Lysozyme supplementation enhances LC-PUFA synthesis, favoring DHA accumulation in low-dose groups (Mus1) and EPA/DHA precursor enrichment in both lysozyme-supplemented diets groups. These results align with the observed improvements in fish muscle nutritional quality. This enhancement is possibly due to lysozyme's modulation of gut microbiota composition and activity, thus, potentially increasing the production of short-chain fatty acids (SCFAs), known to influence host lipid metabolism and gene expression through fatty acid desaturase and elongase [66, 67]. Furthermore, lysozyme may reduce chronic inflammation, thereby suppressing LC-PUFA synthesis pathways. It could also directly interact with lipids to influence digestion, absorption, or cellular uptake [68]. These shifts in LC-PUFA profiles align with the observed improvements in the nutritional quality of the fish muscle.

As depicted in Figure 5a and Supporting Information: Figure S2, Mus2 displayed upregulation of genes associated with the intestinal immune network for IgA production. The mucosal-associated lymphoid tissues (MALTs), including skin-associated lymphoid tissue (SALT), gill-associated lymphoid tissue (GIALT), gut-associated lymphoid tissue (GALT), and nasopharynx-associated lymphoid tissue (NALT) [69], are crucial for preventing pathogen invasion during early infection stages in fish (Figure 6). Lysozyme, one of the most studied innate immune components in fish, lyses bacteria by degrading the peptidoglycan layer of their cell walls [70]. Naturally present in mucus, lymphoid tissues, plasma, and other fluids, lysozyme is widely expressed across various tissues. Dietary lysozyme supplementation alters the fish intestinal microbial community, influencing growth, digestion, immunity, disease resistance, and the development and function of the immune response [71]. Gut microbiota are involved in recruiting and developing immune cells and forming intestinal lymphoid tissue [72]. Furthermore, microbiota influence GALT development by providing essential signals, such as cytokines, which recruit macrophages into the intestine and GALT [69]. Cytokines, key modulators of both innate and adaptive immune responses, include pro-inflammatory types like IL-1β, TNF-α, and IL-6, which are commonly studied immune-regulatory genes in fish [70]. Consequently, lysozyme-supplemented diets may alter the expression of IL-1β, TNF-α, and IL-6, thereby influencing the immune responses of yellowfin seabream.

## 6. Conclusion

This investigation demonstrates that dietary lysozyme supplementation significantly enhances muscle quality and immune function in yellowfin seabream (*Acanthopagrus latus*), xproviding actionable strategies for commercial aquaculture. Our findings reveal that both lysozyme-supplemented diets (Mus1:100 mg/kg lysozyme-supplemented diets; Mus2:200 mg/kg lysozyme-supplemented diets) improved textural properties (hardness, adhesiveness, and gumminess) and increased ash/carbohydrate content compared to the control (Mus3). Metabolite profiling further identified dose-specific benefits: Mus1 fish elevated omega-3 fatty acids (EPA/DHA) and beneficial organic acids (citric and malic acid), enhancing nutritional value for premium markets, while Mus2 fish boosted mucosal immunity and reduced fat deposition, improving disease resilience and product leanness.

For operations targeting value-added, nutritionally enriched seafood, we recommend prioritizing low-dose lysozyme (100 mg/kg lysozyme-supplemented diets, Mus1) to optimize lipid profiles and organic acid content of fish flesh. Conversely, for farms balancing texture improvements with disease resistance in high-density systems, medium-dose supplementation (200 mg/kg lysozyme-supplemented diets, Mus2) is advised to enhance fillet firmness and immune function while minimizing fat deposition. Implementing these tailored strategies can reduce antibiotic reliance, differentiate products in competitive markets, and improve harvest yields. Further refinement of delivery methods may maximize return on investment for yellowfin seabream producers.

## Figures and Tables

**Table 1:** Muscle components of yellowfin seabream (*Acanthopagrus latus*) fed the experimental diets.

Sample	Moisture (g/100 g)	Ash (g/100 g)	Crude Protein (g/100 g)	Crude Fat (g/100 g)	Carbohydrate (g/100 g)
Mus1	73.83 ± 0.12^b^	2.97 ± 0.06^b^	19.83 ± 0.15^a^	3.00 ± 0.10^a^	0.37 ± 0.15^a^
Mus2	73.90 ± 0.20^b^	3.27 ± 0.06^a^	19.73 ± 0.35^a^	2.77 ± 0.12^a^	0.33 ± 0.21^a^
Mus3	74.47 ± 0.21^a^	2.83 ± 0.06^c^	19.60 ± 0.17^a^	2.87 ± 0.06^a^	0.27 ± 0.12^a^

*Note:* Determinations are reported on a wet basis (g/100 g). Values represent mean ± standard deviation (SD) (*n* = 3). The three groups of fish include Mus1 (basal feeds supplemented with 100 mg/kg lysozyme), Mus2 (basal feeds supplemented with 200 mg/kg lysozyme), and Mus3 (basal feeds without lysozyme) designed as a control group. Within the same row, values with different superscript letters denote significant differences (*p*  < 0.05).

**Table 2:** Texture properties of yellowfin seabream (*Acanthopagrus latus*) fed the experimental diets.

Sample	Hardness (N)	Adhesiveness	Cohesiveness	Gumminess (N)	Springiness (mm)	Chewiness (mJ)	Resilience
Mus1	23.82 ± 14^a^	−0.30 ± 0.84^a^	0.28 ± 0.21^b^	23.10 ± 10^a^	4.47 ± 4.4^a^	0.94 ± 4.1^a^	0.17 ± 0.6^ab^
Mus2	28.36 ± 11^a^	−0.30 ± 0.51^a^	0.28 ± 0.23^b^	27.67 ± 18^a^	4.86 ± 4.2^a^	0.77 ± 4.3^a^	0.10 ± 0.6^b^
Mus3	13.18 ± 10^b^	−0.10 ± 0.16^b^	0.39 ± 0.22^a^	13.48 ± 9.7^b^	3.67 ± 2.0^a^	1.92 ± 1.7^a^	0.41 ± 0.5^a^

*Note:* Values represent mean ± standard deviation (SD) (*n* = 6). The three groups of fish include Mus1 (basal feeds supplemented with 100 mg/kg lysozyme), Mus2 (basal feeds supplemented with 200 mg/kg lysozyme), and Mus3 (basal feeds without lysozyme) designed as a control group. Within the same row, values with different superscript letters denote significant differences (*p*  < 0.05).

## Data Availability

The data that support the findings of this study are available from the corresponding author upon reasonable request.

## Supporting Information

Supporting Information Figure S1: KEGG analysis of differentially enriched metabolic pathways and metabolites between Mus1 and Mus3 groups in yellowfin seabream (*Acanthopagrus latus*) muscle tissue. (a) Enrichment analysis of the top 20 differentially enriched metabolic pathways, (b) Differential abundance scores of the top 20 pathways, (c) Variable importance in projection (VIP) scores of the top 30 upregulated and downregulated metabolites. Figure S2: KEGG analysis of differentially enriched metabolic pathways and metabolites between Mus2 and Mus3 groups in yellowfin seabream (*Acanthopagrus latus*) muscle tissue. (a) Enrichment analysis of the top 20 differentially enriched metabolic pathways, (b) Differential abundance scores of the top 20 pathways, (c) VIP scores of the top 30 upregulated and downregulated metabolites. Figure S3: KEGG analysis of differentially enriched metabolic pathways and metabolites between Mus1 and Mus2 groups in yellowfin seabream (*Acanthopagrus latus*) muscle tissue. (a) Enrichment analysis of the top 20 differentially enriched metabolic pathways, (b) Differential abundance scores of the top 20 pathways, (c) VIP scores of the top 30 upregulated and downregulated metabolites.
