# Supplementary material for: Effects of Lysozyme-Supplemented Diets on Muscle Texture and Metabolite Profiles in Yellowfin Seabream (Acanthopagrus latus)
Source: Aquac Nutr. 2025 Nov 19;2025:9977656. doi: 10.1155/anu/9977656 (PMC12657072; doi:10.1155/anu/9977656)
Supplement: Supporting Information — Figure S1: KEGG analysis of differentially enriched metabolic pathways and metabolites between Mus1 and Mus3 groups in yellowfin seabream (Acanthopagrus latus) muscle tissue. (a) Enrichment analysis of the top 20 differentially enriched metabolic pathways, (b) Differential abundance scores of the top 20 pathways, (c) Variable importance in projection (VIP) scores of the top 30 upregulated and downregulated metabolites. Figure S2: KEGG analysis of differentially enriched metabolic pathways and metabolites between Mus2 and Mus3 groups in yellowfin seabream (Acanthopagrus latus) muscle tissue. (a) Enrichment analysis of the top 20 differentially enriched metabolic pathways, (b) Differential abundance scores of the top 20 pathways, (c) VIP scores of the top 30 upregulated and downregulated metabolites. Figure S3: KEGG analysis of differentially enriched metabolic pathways and metabolites between Mus1 and Mus2 groups in yellowfin seabream (Acanthopagrus latus) muscle tissue. (a) Enrichment analysis of the top 20 differentially enriched metabolic pathways, (b) Differential abundance scores of the top 20 pathways, (c) VIP scores of the top 30 upregulated and downregulated metabolites. [file 9977656.f1.docx]

**Supplementary Materials**

**Exploring the Effects of Lysozyme Feed on Muscle Texture and Metabolite Profiles in Yellowfin Seabream (*Acanthopagrus latus*)**

Wenmeng He^1^, Genmei Lin^2,3^*, Lu-jing Gan^4^, Min Chen^2^, Yinjun Ye^2^, Huixin Zhao^2^, Jianbo Yao^2^, Xuan Cao^2^, Kaidiriye Kaisaier^2^, Kaizhuo Cai^2^, and Yitao Zhou^2^

1. Department of Life Science, Faculty of Science and Technology, Beijing Normal -Hong Kong Baptist University, Zhuhai, Guangdong, China.
2. School of Marine Sciences, Sun Yat-sen University, Zhuhai, Guangdong, China.
3. Southern Marine Science and Engineering Guangdong Laboratory (Zhuhai), Zhuhai, Guangdong, China.
4. Department of Food Science and Engineering, School of Materials and Environment, Beijing Institute of Technology University, Zhuhai, Guangdong, China.

*Corresponding author at

Dr. Genmei Lin

Email: lingm5@mail.sysu.edu.cn

Postal address: School of Marine Sciences, Sun Yat-sen University, Zhuhai 519080, China

**Table of contents**

**Supplementary Figures**

**Figure S1**. KEGG analysis of differentially enriched metabolic pathways and metabolites between Mus1 and Mus3 groups in yellowfin seabream (*Acanthopagrus latus*) muscle tissue. (a) Enrichment analysis of the top 20 differentially enriched metabolic pathways, (b) Differential abundance scores of the top 20 pathways, (c) Variable importance in projection (VIP) scores of the top 30 upregulated and downregulated metabolites.

**Figure S2**. KEGG analysis of differentially enriched metabolic pathways and metabolites between Mus2 and Mus3 groups in yellowfin seabream (*Acanthopagrus latus*) muscle tissue. (a) Enrichment analysis of the top 20 differentially enriched metabolic pathways, (b) Differential abundance scores of the top 20 pathways, (c) VIP scores of the top 30 upregulated and downregulated metabolites.

**Figure S3**. KEGG analysis of differentially enriched metabolic pathways and metabolites between Mus1 and Mus2 groups in yellowfin seabream (*Acanthopagrus latus*) muscle tissue. (a) Enrichment analysis of the top 20 differentially enriched metabolic pathways, (b) Differential abundance scores of the top 20 pathways, (c) VIP scores of the top 30 upregulated and downregulated metabolites.

**Figure S1**. KEGG analysis of differentially enriched metabolic pathways and metabolites between Mus1 and Mus3 groups in yellowfin seabream (*Acanthopagrus latus*) muscle tissue. (a) Enrichment analysis of the top 20 differentially enriched metabolic pathways, (b) Differential abundance scores of the top 20 pathways, (c) Variable importance in projection (VIP) scores of the top 30 upregulated and downregulated metabolites.

**Figure S2**. KEGG analysis of differentially enriched metabolic pathways and metabolites between Mus2 and Mus3 groups in yellowfin seabream (*Acanthopagrus latus*) muscle tissue. (a) Enrichment analysis of the top 20 differentially enriched metabolic pathways, (b) Differential abundance scores of the top 20 pathways, (c) VIP scores of the top 30 upregulated and downregulated metabolites.

**Figure S3**. KEGG analysis of differentially enriched metabolic pathways and metabolites between Mus1 and Mus2 groups in yellowfin seabream (*Acanthopagrus latus*) muscle tissue. (a) Enrichment analysis of the top 20 differentially enriched metabolic pathways, (b) Differential abundance scores of the top 20 pathways, (c) VIP scores of the top 30 upregulated and downregulated metabolites.
